# 

**Published:** 1908-05-30

**Authors:** 


					-SURGLGM GflHslRAL''- OFFICE
T*l?graphlc Address * ? o -.-..THEj MODERN Telephone Number s
?apedalo T? %T a r 2*34 Gerrard.
London. MEDICAL JOURNAL.
_No. 1135. voi.^juLiv.0' " Saturday,
May 30th, 1908.
PRICE THREEPENCE.

				

